# Accumulation and cellular toxicity of aluminum in seedling of *Pinus massoniana*

**DOI:** 10.1186/s12870-014-0264-9

**Published:** 2014-09-30

**Authors:** Huanhuan Zhang, Ze Jiang, Rong Qin, Huaning Zhang, Jinhua Zou, Wusheng Jiang, Donghua Liu

**Affiliations:** Tianjin Key Laboratory of Animal and Plant Resistance, College of Life Sciences, Tianjin Normal University, Tianjin, 300387 PR China; School of Life Science, South China Normal University, Guangzhou, Guangzhou 510631 PR China

**Keywords:** *Pinus massoniana*, Aluminum (Al), Cell division, Microtubules, Nucleolar organizing region, Nucleolar proteins

## Abstract

**Background:**

Masson pine (*Pinus massoniana*) is one of the most important timber species with adaptable, fast growing, versatile advantages in southern China. Despite considerable research efforts, the cellular and molecular mechanisms of A1 toxicity and resistance in *P. massoniana* are still poorly understood. The effects of Al on uptake and translocation of Al and other minerals, cell division and nucleolus in *P. massoniana* were investigated.

**Results:**

The results indicated that Al accumulated mainly in the roots, and small amounts were transported to aboveground organs. In the presence of Al, the contents of Mg and Fe in stems increased and decreased in roots. Accumulation of Mn in the organs was inhibited significantly. Evidence from cellular experiments showed that Al had an inhibitory effect on the root growth at all concentrations (10^−5^ – 10^−2^ M) used. Chromosome fragments, chromosome bridges, C-mitosis and chromosome stickiness were induced during mitosis in the root tip cells. Al induced the formation of abnormal microtubule (MT) arrays, consisting of discontinuous wavy MTs or short MT fragments at the cell periphery. MT organization and function of the mitotic spindle and phragmoplast were severely disturbed. The nucleolus did not disaggregate normally and still remained its characteristic structure during metaphase. Nucleolar particles containing argyrophilic proteins were accumulated and leached out from the nucleus to the cytoplasm. Evidence confirmed that these proteins contained nucleophosmin (B23), nucleolin (C23) and fibrillarin. Western immunoblot analysis revealed that the contents of three nucleolar proteins increased significantly.

**Conclusion:**

Based on the information provided in this article, it is concluded that root tips of plants are the most sensitive organ to environmental stresses and the accumulation of Al ions primarily is in roots of *P. massoniana*, and small amounts of Al are transported to aboveground. Root apical meristems play a key role in the immediate reaction to stress factors by activating signal cascades to the other plant organs. Al induces a series of the cellular toxic changes concerning with cell division and nucleolus. The data presented above can be also used as valuable and early markers in cellular changes induced by metals for the evaluation of metal contamination.

## Background

Aluminium (Al) ranks third in abundance among the Earth’s crust elements, after oxygen and silicon, and is the most abundant metallic element [1,2]. Al is a ubiquitous element without a known, specific and biological function in plant metabolism [3]. However, the metal is considered to be a major growth-limiting factor particularly in acid soils (pH < 5.0), which are estimated to be approximately 30–40% of arable lands in the world [4]. Once the pH decreases below 5.0, Al is solubilized into a phytotoxic form, mainly as Al^3+^ from nonphytotoxic silicate or oxide forms which restricts plant growth [5].

It is well known that Al, for most crops, is a serious constraint, although some crops (e.g., pineapple and tea) are considered to be tolerant to high levels of exchangeable Al. Species and genotypes within species greatly differ in their tolerance to Al [2]. Investigations on the toxicity and resistance mechanisms have often been performed taking physiological and genetic basis of resistance into consideration [6,7]. Some investigations indicate that Al uptake is limited mainly to the root system, where it accumulates predominantly in the epidermis and the outer cortex [8,9]. And the others demonstrate that some plant species, such as some species native to the region of central Brazil, can accumulate considerable amounts of Al in their shoots [9,10]. Due to its importance in limiting agricultural and forest productivity, there have been numerous studies that describe the toxic effects of Al on plant root growth and physiology [11,12]. Probing root meristem as a plant bioassay system for Al toxicity testing has been suggested since many plants are known to be injured by Al under natural and experimental exposure conditions. It has been well known that Al toxicity is performed primarily by inhibition of root growth [13], and the root meristem is one of the most sensitive sites to Al toxicity [14].

Masson pine (*Pinus massoniana*) is one of the most important timber species with adaptable, fast growing, versatile advantages in southern China [15]. *P. massoniana* occupies a very important position in China’s forestry development and forest resources cultivation, and has expanded rapidly to reach an estimated area of 5.7 million hectares [16]. It was reported that due to heavy chemical fertilization the soil pH in the major Chinese crop-production areas declined significantly from the 1980s to the 2000s [17]. Al toxicity due to soil acidification has become the main reason for the decline of the forest [18]. Despite considerable research efforts, the cellular and molecular mechanisms of A1 toxicity and resistance in *P. massoniana* are still poorly understood.

The effect of Al on uptake and translocation of Al and other minerals (Fe, Mn and Mg), cell division and nucleolus in *P. massoniana* were carried out in order to understanding the mechanisms of Al-induced toxicity.

## Results

### Al accumulation and its effect on other minerals

#### Al accumulation

Al uptake and accumulation in roots, leaves and shoots of *P. massoniana* varied depending on Al concentration. The Al contents increased significantly (*p* < 0.05) with increasing Al concentration in the nutrient solutions (Table 1). The accumulation of Al primarily was in roots, and small amounts of Al were transported to stems and leaves. Levels of Al in *P. massoniana* treated with 10^−5^ M to 10^−3^ M Al were in the order: roots > leaves > stems, while contents in the treatment group exposed to 10^−2^ M Al were in the order as follows: roots > stems > leaves. The over ground parts (stems and leaves)/roots ratios at 10^−4^ M and 10^−3^ M Al after 40 d of Al treatment were 8.6% and 5%, respectively. However, the ratios at 10^−5^ M and 10^−2^ M Al were higher: they were 30% and 32.1%, respectively.

**Table 1:** **Al content in different organs of** ***Pinus massoniana*** **L. exposed to different concentrations after 40 d treatment**

**Treatment (Al, M)**	**Organs (μg/g DW ± SE)**		
	**Roots**	**Leaves**	**Stems**
Control	23.02 ± 0.22e	18.33 ± .021e	9.38 ± 0.26d
10^−5^	373.75 ± 4.82d	87.83 ± 9.99d	24.25 ± 1.84d
10^−4^	3384.66 ± 19.82c	171.25 ± 10.85c	120.55 ± 1.58c
10^−3^	10024.62 ± 83.66b	278.47 ± 1.91b	226.56 ± 3.34b
10^−2^	14712.89 ± 36.94a	1383.98 ± 4.41a	3340.84 ± 12.07a

Values followed by different letters are significantly different (*P* < 0.05). Vertical bars denote SE (n = 3).

#### Effects of Al on levels of Mg, Fe and Mn

*P. massoniana* seedlings exposed to Al solution substantially affected the uptake and distribution of Mg, Fe and Mn in plants. The results indicated that the contents of Mg and Fe increased in stems of *P. massoniana* seedlings and decreased in roots with increasing Al. While the contents of Fe in the roots and the leaves decreased. Besides, uptake and accumulation of Mn in the organs were inhibited significantly (P < 0.05) under Al stress (Table 2).

**Table 2:** **Effects of Al on accumulation of Mg, Fe and Mn in roots, stems and leaves of** ***Pinus massoniana***

**Element**	**Treatment (Al, M)**	**Organs (μg/g DW ± SE)**		
		**Roots**	**Roots**	**Roots**
Mg	Control	1550.67 ± 11.07a	1169.62 ± 3.15e	2777.18 ± 4.15a
	10^−5^	1533.11 ± 5.06a	1331.27 ± 0.55d	2734.00 ± 14.40ab
	10^−4^	1378.91 ± 11.64b	1421.89 ± 3.54c	2661.81 ± 20.09c
	10^−3^	1196.43 ± 9.65c	2088.24 ± 4.17b	2719.36 ± 9.03b
	10^−2^	1158.62 ± 8.18d	2710.64 ± 16.92a	2716.34 ± 27.94b
Fe	Control	990.08 ± 9.42a	49.59 ± 7.39c	207.84 ± 6.50a
	10^−5^	895.83 ± 2.94b	53.58 ± 0.55c	194.45 ± 0.83b
	10^−4^	725.19 ± 3.11c	87.92 ± 2.39b	178.04 ± 0.63c
	10^−3^	519.29 ± 5.25d	92.22 ± 2.30b	116.23 ± 0.93e
	10^−2^	302.56 ± 2.79e	138.34 ± 1.30a	141.58 ± .69d
Mn	Control	302.00 ± 2.14a	10.05 ± 0.12a	125.07 ± 0.08a
	10^−5^	165.67 ± 0.64b	9.24 ± 0.21b	59.53 ± 0.41c
	10^−4^	151.99 ± 0.55c	8.28 ± 0.24c	61.56 ± 0.14b
	10^−3^	12.81 ± 0.29d	6.75 ± 0.10d	20.56 ± 0.15d
	10^−2^	9.13 ± 0.08e	5.18 ± 0.19e	11.81 ± 0.14e

Values followed by different letters are significantly different (*P* < 0.05). Vertical bars denote SE (n = 3).

### Effects of Al on cell division and nucleoli

#### Effects of Al on root growth

**Figure 1:**
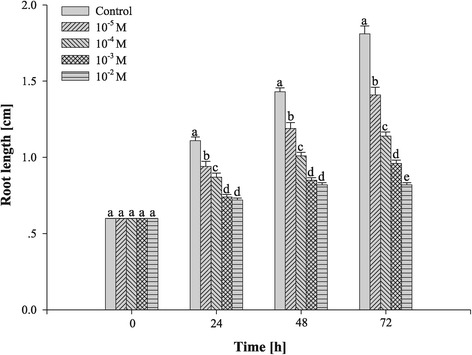
**Effects of different concentrations of Al on root length of** ***P. massoniana*** **stressed for 24, 48 and 72 h.** Values with different letters differ significantly from each other (n = 10, *P* < 0.05).

**Figure 2:**
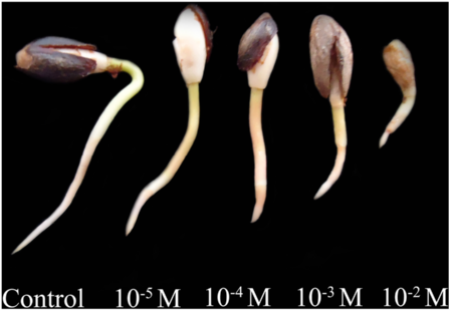
**Effects of different concentrations of Al on seedling growth of** ***P. massoniana*** **during the whole treatment (72 h).**

The effects of Al on the root growth of *P. massonian* varied with the different concentrations of aluminum chloride solutions used (Figures 1 and 2). Al had an inhibitory effect on the root growth at all concentrations (10^−5^–10^−2^ M) used during the entire treatment (72 h). At 10^−3^ –10^−2^ M Al, the root length was strongly inhibited after 24 h of treatment.

#### Effects of Al on chromosome morphology

**Figure 3:**
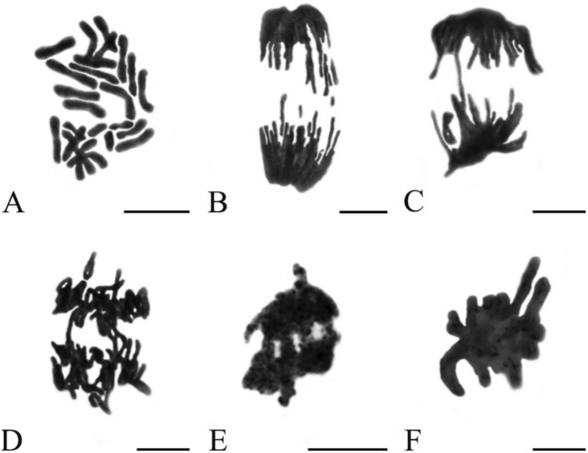
**Effects of Al on root tip cells division of** ***P. Massoniana*** **. A**. C-mitosis (10^−5^ M Al, 24 h). **B**. Chromosome fragments (10^−5^ M Al, 24 h). **C-D**. Chromosome bridges (**C-D**. 10^−4^ M Al, 72 h). **E**. Sticky chromosome bridges (**E**. 10^−3^ M Al, 72 h). **F**. Chromosome stickiness (10^−2^ M Al, 48 h). Scale bar = 10 μm.

The standard types of aberrant chromosomes (modified Allium test introduced by Fiskesjö [19]), were observed in the root tip cells of *P. massoniana* after treatment with Al. The toxic effects of Al on chromosome behavior in root tips of *P. massoniana* varied with the different Al concentrations and the treatment time (Figure 3). Several types of chromosomal aberrations were observed when compared with control. At low concentration (10^−5^ M Al), C-mitosis induced by Al in the present investigation is major type of chromomsomal aberrations and the highly condensed chromosomes were scattered randomly in root tip cells (Figure 3A). Chromosome fragments in some root tip cells were also observed at 10^−5^ M Al (Figure 3B). Anaphase bridges involving one or more chromosomes (Figure 3C–D) were found after the treatment with 10^−4^ M Al. Chromosome stickiness consisted of anaphase sticky bridges (10^−3^ M Al) (Figure 3E) and metaphase sticky chromosomes (10^−2^ M Al) (Figure 3F), which is major type of chromosomal aberrations at high concentration of Al. This type of toxic effect is most likely irreversible, which probably led to cell death.

#### Effects of Al on the organization of MT cytoskeleton

**Figure 4:**
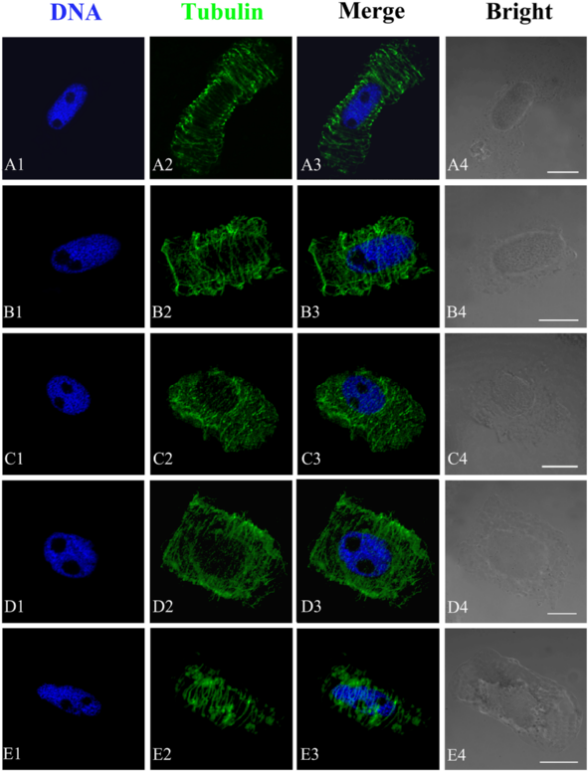
**Effects of different concentrations of Al on the organization of microtubule cytoskeleton in root tip cells of** ***P. massoniana*** **.** DNA staining with DAPI (**A1**–**E1**, blue), tubulin immunolabeling (**A2**–**E2**, green), merged images (**A3**–**E3**) and bright field images (**A4**–**E4**) in the same single optical section obtained with the confocal laser scanning microscope. Bar = 10 μm for all figures. **A**. Interphase cells. The nucleus is surrounded by numerous cortical MTs that are orientated transversely to the long cell axis. **B**. Showing aberrant cortical MTs with slightly skewed wavy (10^−5^ M Al, 24 h). **C–D**. Showing numerous interconnected and short MT fragments distributed randomly at the cell periphery (10^−4^ M Al, 48 h). **E**. Showing MT stickiness (10^−3^ M Al, 48 h).

In controls, cortical microtubules (MTs) of meristematic cells were very abundant during interphase. They were found roughly parallel to each other and were oriented perpendicular to the primary axis of cell expansion (Figure 4A). Al caused the changes in the organization of microtubular cytoskeleton in *P. massoniana* cells. Some cells displaying aberrant cortical MTs were found after 24 h treatment with 10^−5^ M Al. In these cells MT organization was traversed by slightly skewed wavy (Figure 4B). When cells exposed to 10^−4^ M Al for 48 h, the cortical MTs of some cells lost their transverse organization. Instead, they were randomly oriented, often discontinuous and form numerous short MT fragments of different size at the cell periphery (Figure 4C–D). MT stickiness was observed in some cells treated with 10^−3^ M Al for 48 h. The proportion of abnormal MTs increased depending on Al concentrations and duration of treatment. Evidences above suggested that Al damaged structure of cortical MTs.

**Figure 5:**
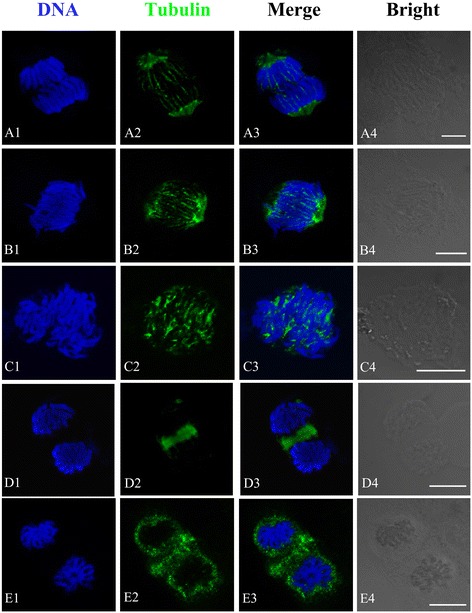
**Effects of different concentrations of Al on spindle MTs in mitosis in root tip cells of** ***P. massoniana*** **.** DNA staining with DAPI (**A1**–**D1**, blue), tubulin immunolabeling (**A2**–**D2**, green), merged images (**A3**–**D3**) and bright field images (**A4**–**D4**) in the same single optical section obtained with the confocal laser scanning microscope. Bar = 10 μm for all figures. **A**. Showing anaphase chromosome and spindles in control cell. **B**. Showing sticky spindle and MT fragments in anaphase (10^−4^ M Al, 24 h). **C**. Showing depolymerized spindle MTs and chromosome stickiness (10^−3^ M Al, 48 h). **D**. Showing normal phragmoplast (Control). **E**. Showing depolymerized phragmoplast (10^−3^ M Al, 48 h).

In metaphase and anaphase cells of control, the typical mitotic spindles is that spindle MTs become oriented into a bipolar array whose dyad axis divided the structure into two half spindles, and sister chromatids were segregated by moving them to opposite poles (Figure 5A). The changes of MT cytoskeleton induced by Al were closely related to chromosome aberrations (anaphase bridges, C-mitosis and chromosome stickiness) during mitosis. The significant changes in MT cytoskeleton were noted in the cells exposed to 10^−4^ M Al after 24 h, revealing some sticky spindle MTs split into discontinuous MT fragments in the procession of moving sister chromatids to opposite poles (Figure 5B). With increasing Al concentration and duration of treatment, spindle MT arrays were mostly depolymerized in some cells, resulting in the formation of chromosome stickiness (Figure 5C). In control cells, phragmoplast expanded centrifugally until it contacted the parent cell walls and daughter chromosomes were reorganized into new nuclei (Figure 5D). Phragmoplast could not be formed due to damage of MTs of the mitotic spindle in anaphase cells treated with 10^−3^ M Al after 48 h (Figure 5E).

#### Effects of Al on nucleolar cycle during mitosis

**Figure 6:**
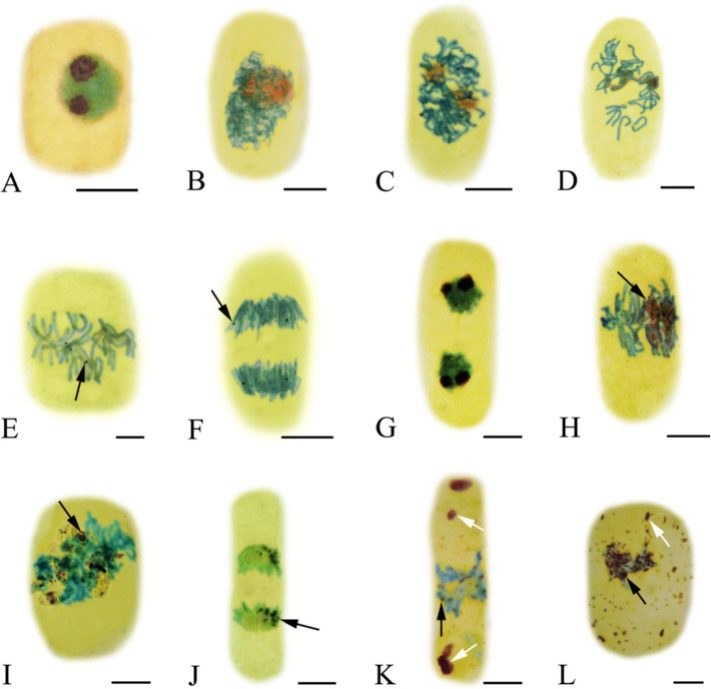
**Effects of Al on NORs in root tip cells of** ***P. massoniana*** **during mitosis (Black arrowheads show NORs; White arrowheads show silver stained materials). A–I**. Normal mitotic process. **A–C**. Showing decondensed chromatin fibers around the nucleoli. **D**. Showing decreased nucleoli in size. **E**. Showing filamentous NORs at late prophase. **F**. Showing NORs on metaphase chromosome. **G**. Showing NORs migration with the chromosomes to the poles at anaphase. **H–L**. Mitotic process under Al stress. **H–J**. Nucleoli still existed after the treatment with Al during metaphase (**H**: 10^−5^ M Al, 72 h; **I**: 10^−4^ M Al, 24 h; **J**: 10^−3^ M Al, 24 h ). **K–L**. Showing some small NORs localized on sticky chromsomes and similar silver stained particles distributed in cytoplasm (**K**: 10^−4^ M Al, 72 h; **L**: 10^−3^ M Al, 72 h). Scale bar = 10 μm.

The nucleolar cycle of silver-impregnated *P. massoniana* cells was examined by means of light microscopy. Normally, the nucleoli in interphase nuclei impregnated with silver showed strong staining (Figure 6A). Then the prophase decondensed chromatin fibers appeared gradually and were around the nucleoli (Figure 6B–C). During prometaphase-metaphase, the nucleoli became small in size (Figure 6D). The nucleoli disappeared completely in their characteristic structures and nucleolar organizing regions (NORs) were localized on metaphase chromosomes (Figure 6E). NORs were migrated with the chromosomes to the poles at anaphase (Figure 6F). The newly forming nucleoli around the NORs were rebuilt at telophase (Figure 6G). Mitosis was completed. After the treatment with Al, the abnormal phenomena of the nucleolar cycle during mitosis were examined in some cells. Firstly, the nucleoli were not disaggregated normally and still remained their characteristic structures during metaphase (Figure 6H–I) and anaphase (Figure 6J), which was called persistent nucleoli. Secondly, some small NORs were localized on sticky chromosomes and more similar silver stained particles were distributed in cytoplasm (Figure 6K–L).

#### Effects of Al on nucleoli

**Figure 7:**
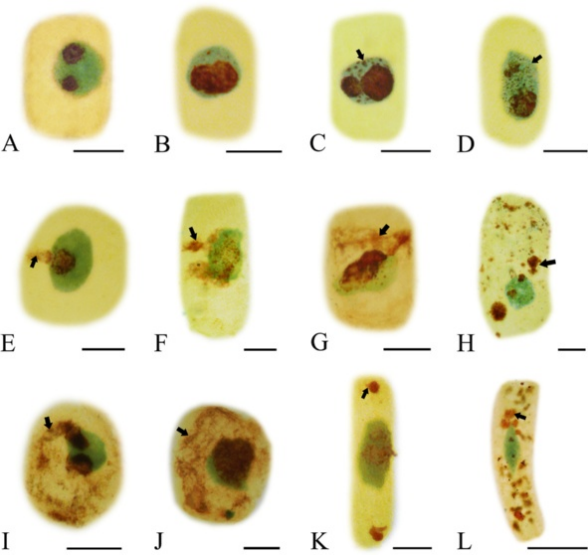
**Effects of different concentrations of Al on nucleoli in root tip cells of** ***P. massoniana*** **.** Arrowhead shows argyrophilic proteins. **A**. Control cells. **B**. Showing the irregular nucleolus (10^−5^ M Al, 72 h). **C**. Some particles containing argyrophilic proteins scattered in the nucleus (10^−4^ M Al, 24 h). **D**. Large amounts of argyrophilic proteins in nucleus with prolonging treatment time (10^−4^ M Al, 48 h). **E–G**. Argyrophilic proteins leaching from the nucleus to the cytoplasm and more and more argyrophilic proteins accumulated in the cytoplasm with prolonging the duration of treatment (**E**.10^−4^ M Al, 48 h; **F-G**. 10^−4^ M Al, 72 h). **H–J**. Showing the argyrophilic proteins enclosed the nucleus, and accumulated in the cytoplasm and occupied nearly the whole cytoplasm (**H**. 10^−3^ M Al, 72 h; **I**. 10^−2^ M Al, 72 h; **J**. 10^−2^ M Al, 72 h). **K**. In long cells, the argyrophilic proteins gathered at the cell ends (10^−2^ M Al, 24 h). **L**. Argyrophilic proteins scattered in the nucleus, appearing small in size and weak in silver staining reaction (10^−2^ M Al, 24 h). Scale bar =10 μm.

Normally, the nucleus of *P. massoniana* contains 1 to 2 nucleoli (Figure 7A). The toxic effects of Al on nucleoli varied with the concentration and the treatment time. At low concentration (10^−5^ M Al), nucleoli were irregularly swollen in most of the root tip cells (Figure 7B). Some tiny particles containing argyrophilic proteins were scattered in the nucleus of root tip cells exposed to 10^−4^ M Al for 24 h (Figure 7C). Large amounts of the tiny particulates were observed with prolonged the treatment time (Figure 7D). In Figure 7E, the particles were accumulated and leached out from the nucleus to the cytoplasm after 10^−4^ M Al treatment for 48 h. The phenomenon was also observed in the cells exposed to 10^−3^ M Al after 72 h treatment. The amount of this particulate material increased progressively in cytoplasm (Figure 7F–G) and nearly occupied the whole cytoplasm when the Al concentration was increased to 10^−2^ M (Figure 7H–J). In long cells, the nucleolar materials were extruded from the nucleus into the cytoplasm and gathered at the cell ends, and large rod-like structures were formed (Figure 7K). The nucleolar remains in the nucleus became small in size and weak in silver staining reaction (Figure 7L).

#### Translocation of the three major nucleolar proteins in relation to Al treatment

Immunofluorescence localizations of B23, C23 and fibrillarin were performed in the present investigation. The antibodies used could produce positive reactions with three nucleolar proteins above. There were obvious toxic effects on the three nucleolar proteins in the root tip cells of *P. massoniana* exposed 10^−2^ M Al treatment when compared with control cells.

**Figure 8:**
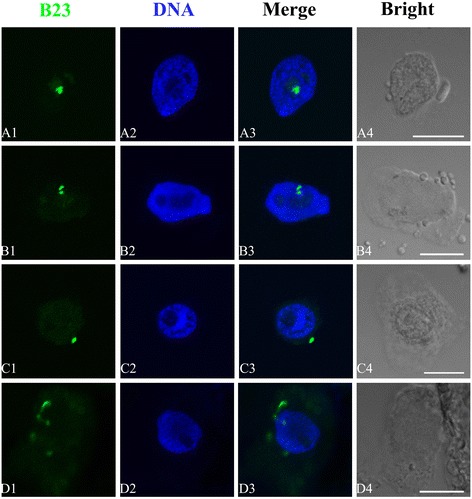
**Effects of different concentrations of Al on the translocation of B23.** Simultaneous location of B23 after the reaction with primary anti-B23 antibody and secondary antibody conjugated with FITC (green) and DNA after the reaction with DAPI (blue) in the same single optical section obtained with the confocal scanning laser microscopy. **A1–D1**, B23 detection; **A2–D2**, DNA detection; **A3–D3**, Merged image; **A4–D4**, Bright field image of the cells. **A1–A3**, B23 was localized in nucleolus in control cells. **B1-B3**, Showing that B23 was migrated from nucleolus to nucleoplasm in the cells exposed to10^−4^ M Al for 72 h. **C–D**, Showing that B23 was scattered in cytoplasm of the cells exposed to 10^−2^ M Al for 72 h and the intensity of B23 signals increased in cytoplasm. Scale bar =10 μm.

B23 was appeared green in colour by blue light after tagging with FITC under immunofluorescent microscopy. The images in Figure 8 obtained from confocal microscopy showed that B23 signals monitored by the anti-B23 antibody were all distributed in nucleolus in control cells (Figure 8A1–A3). After 10^−2^ M Al treatment for 72 h, firstly, B23 signals were transferred from nucleolus to nucleoplasm (Figure 8B1–B3). Then large amounts of B23 signals were observed in cytoplasm, and localized around the nucleus in varying degrees (Figure 8C–D).

**Figure 9:**
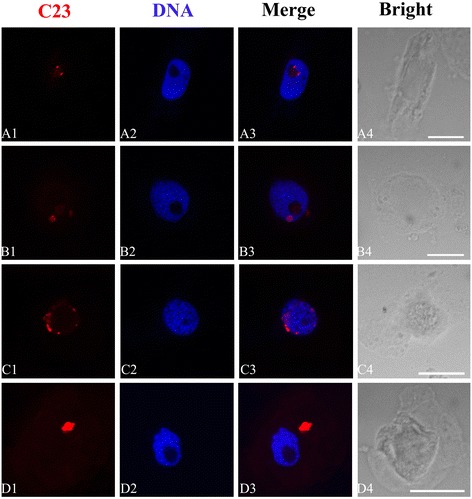
**Effects of different concentrations of Al on the translocation of C23.** Simultaneous location of C23 after the reaction with primary anti-C23 antibody and secondary antibody conjugated with TRITC (red) and DNA after the reaction with DAPI (blue) in the same single optical section obtained with the confocal scanning laser microscopy. **A1–D1**, C23 detection; **A2–D2**, DNA detection; **A3–D3**, Merged image; **A4–D4**, Bright-field image of the cells. **A1-A3**, Showing that C23 was distributed in nucleolus in control cells. **B-C**, Showing that C23 was migrated from nucleolus to nucleoplasm and cytoplasm in the cells exposed to10^−4^ M Al for 72 h. **D1–D3**, Showing that in some cells bigger fluorescence signals of C23 appeared and were extruded from nucleolus into cytoplasm after the exposure of 10^−2^ M Al for 72 h. Scale bar =10 μm.

Nucleolar protein C23 was marked with TRITC and produced red fluorescent signal under confocal microscopy. The present investigation proved that red small amount of immunofluorescence spots of C23 were scattered in nucleolus in control cells (Figure 9A1–A3). Exposure of cells to Al (10^−2^ M) for 72 h, the transformation of C23 localization was remarkable when compared with control. More C23 signals were seen in nucleoplasm, and on the way from nucleoplasm to cytoplasm (Figure 9B–C). Besides, the intensity of red fluorescent signal increased in cytoplasm after the treatment with Al (Figure 9D1–D3). The phenomenon was similar to the results of B23.

**Figure 10:**
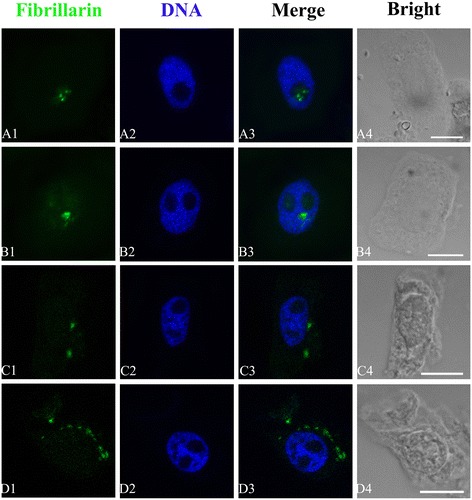
**Effects of different concentrations of Al on the translocation of fibrillarin.** Simultaneous location of fibrillarin after the reaction with primary anti-fibrillarin antibody and secondary antibody conjugated with FITC (green) and DNA after the reaction with DAPI (blue) in the same single optical section obtained with the confocal scanning laser microscopy. **A1–D1**, Fibrillarin detection; **A2–D2**, DNA detection; **A3–D3**, Merged image; **A4–D4**, Bright-field image; **A1–A3**, Showing that fibrillarin was localized in nucleolus in control cells. **B1–B3**, Showing that fibrillarin was transferred from nucleolus to nucleoplasm in the cells treated with 10^−4^ M Al for 72 h. **C1–C3**, Showing that fibrillarin was on the way from nucleus to cytoplasm in the cells treated with 10^−2^ M Al for 72 h. **D1–D3**, Showing that larger amount of fluorescence signals of fibrillarin appeared and was transferred from nucleolus to cytoplasm in the cells exposed to 10^−2^ M Al for 72 h. These signals were close to nucleus and accumulated around the nucleus in varying degrees. Scale bar =10 μm.

Nucleolar protein fibrillarin was also observed using the green fluorescent signal. Normally, green fluorescent signals of fibrillarin appeared in nucleolus in control cells of *P. massoniana* (Figure 10A1–A3). In comparison with control cells, fibrillarin was transferred from nucleolus to nucleoplasm (Figure 10B1–B3), and in some cells it was moved from nucleoplasm to cytoplasm (Figure 10C1–C3) after the treatment of 10^−2^ M Al. Moreover, a great number of bigger green fluorescence spots were scattered in cytoplasm and accumulated around the nucleus (Figure 10D1–D3).

#### Expression of the three major nucleolar proteins in relation to Al treatment

**Figure 11:**
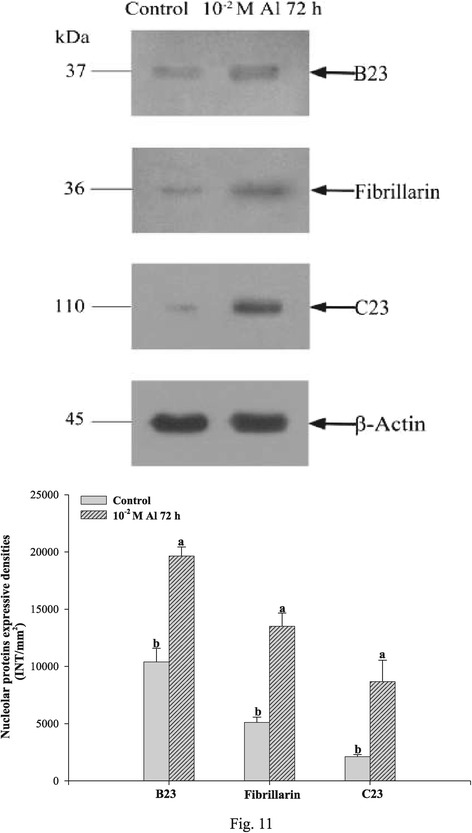
**Effects of Al on the expression of B23, C23 and fibrillarin in the root tip cells of** ***P. massoniana*** **exposed to 10** ^**−2**^ **M Al for 72 h.** The bands were detected by western blotting analysis (upper) and their intensities were measured by “Quantity One” software (lower). Bar indicates standard error (SE). n = 3. *P* < 0.05.

The contents of the three major nucleolar proteins (B23, fibrillarin and C23) in root tip cells of *P. massoniana* L. exposed to 10^−2^ M Al for 72 h were examined by western blotting, raised with specific antibodies. The evidences indicated that levels of the three examined proteins augmented significantly (*P* < 0.05) in comparison with control (Figure 11). The increase of C23 was the most obvious and fibrillarin and B23 were less significant. The phenomena were consistent with the results obtained from indirect immunofluorescent microscopy.

## Discussion

### Al uptake and its effects on mineral elements

Data from the present investigation showed Al was poorly translocated from roots to leaves and stems in *P. massoniana* and accumulated in its leaves and stems much less in 10^−5^ M to 10^−3^ M Al treatment groups, but it accumulated significantly in them exposed to 10^−2^ M Al (Table 1). *P. massoniana* survived as far as it was able to avoid Al accumulation in the shoots. Species and genotypes within species greatly differ in their tolerance to Al [2]. Osaki et al. [20] indicated that some species, especially those native to acidic soils, had shown enhanced growth in the presence of Al, often coinciding with increased leaf phosphorus (P) concentrations. It was reported that Al was essential for the growth of *Melastoma malabathricum* [21]. Jansen et al. [10] proposed that plants with more than 1000 mg Al per kg dry weight in their leaf tissues be termed hyperaccumulators. According to the results here, *P. massoniana* cannot be considered as a hyperaccumulator, althrough the Al content of leaf tissues exposed to 10^−2^ M Al reached the hyperaccumulator standard. The seedling growth of *P. massoniana* was severely inhibited under 10^−2^ M Al stress. *P. massoniana* treated with 10^−5^ to 10^−3^ M Al did accumulate Al in stems and leaves, but could not absorb and accumulate large amounts of Al (Table 1).

Magnesium (Mg) is pivotal for activating a large number of enzymes; hence, Mg plays an important role in numerous physiological and biochemical processes affecting plant growth and development [22]. The results indicated that the contents of Mg and iron (Fe) in stems of *P. massoniana* seedlings increased and decreased in roots with increasing Al (Table 2). Al-induced Mg deficiency in roots may be explained by the fact that the Al and Mg ions compete for membrane transporters and metal-binding sites on enzymes [23], as Al and Mg ions have similar hydrated radius [24]. As a result, Mg ion bind relatively weakly to the negatively charged groups in the root cell wall, so the excess cations such as H^+^ and Al^3+^ present in acid soils can inhibit Mg^2+^ loading into the apoplasm and uptake across the plasma membrane [25]. After uptake, Mg must be released to the xylem for translocation from the roots to the shoots. In the present investigation, Mg content in stems of *P. massoniana* seedlings increases. However, transporters for this process have not been identified. Chen and Ma [26] indicated that based on the RiceXPro database, two CorA-like homologues in rice show high expression in the vascular tissue of root elongation and maturation zones, suggesting their possible role in Mg xylem loading. The transporters for xylem unloading have also not been identified.

Reports on the effects of Al on uptake of manganese (Mn) are conflict (Table 2). Alam [27] indicated that Al could decrease the concentration of Mn in all parts of barley plants except stem, where more Mn concentration was recorded. However, in rice, Mn concentration decreased in plant tops but increased in roots with increasing Al, suggesting that Mn may compete effectively with A1 for root absorption sites [28]. Mariano and Keltjens [29] found that all 10 maize genotypes absorbed Mn in amounts significantly lower than their control plants grown in the absence of Al. These conflicting results arise, in part, from the genetic material used. Data from the present investigation showed that uptake and accumulation of Mn in the organs were inhibited significantly (*P* < 0.05) under Al stress. The results here indicated that the content of Fe in stems of *P. massoniana* seedlings increased with increasing Al, which is consistent with the findings of Alam [28] and Guo et al. [30].

### Toxic effects of Al on cell division

The inhibition of root elongation is the first visible symptom of Al toxicity, although the response of the roots to Al toxicity differs among plant species and even cultivars. In the present study, Al had an inhibitory effect on the root growth at all concentrations (10^−5^–10^−2^ M) used during the entire treatment (72 h) (Figures 1 and 2), suggesting that root cells is a primary target of Al toxicity. The dissociation of the metallic salt AlCl_3_ altered the ionic environment of the cell, which might have led to a physiological change in the nucleoprotein or denaturation of proteins reflected as chromosomal aberrations [31]. Some reports indicated that in sensitive plants, cell division in the root tip meristem was quickly inhibited by Al, resulting in affecting root elongation immediately [32-34].

Chromosome aberrations have been used as a measure of reproductive success and as a method for the detection of possible genetic damage by environmental agents (such as herbicides, insecticides, fungicides and heavy metals) in plants for many years, and can provide both qualitative and quantitative data on the effects [35]. Cytogenetic analysis has also revealed the presence of abnormally dividing cells. Our cytological observations clearly showed that Al had toxic effects on the cell division and induced the four types of chromosomal aberrations, chromosome fragments, chromosome bridges, C-mitosis and chromosome stickiness (Figure 3), which is similar to previous reports described by Liu et al. [36]. The chromosome bridges exhibiting stickiness was found in the present study, which was in agreement with other reports where Al, Cd and Cr (VI) on root tip cell division of *Oryza sativa* [31], *Allium cepa* [37] and *Amaranthus viridis* [38] were investigated. Some studies concerning with the reasons for the formation of stickiness of chromosomes have been reported, such as the increased chromosome contraction and condensation [39], the depolymerization of DNA [40] and partial dissolution of nucleoproteins [41]. This kind of chromosomal aberration, usually being irreversible, reflects highly toxic effects and probably leads to cell death. C-mitosis, first described by Levan [42] in the root tip mitosis of *Allium cepa* as an inactivation of the spindle followed by random scattering of the condensed chromosomes. The c-metaphase we observed in the treated meristems suggests that Al acts on the mitotic spindle apparatus, probably interfering with the polymerization and depolymerization of microtubules [43]. Chromosome bridges or interchromatid connections are formed by chromatin fibers that join sister chromatids at metaphase and hold the chromatids together until late anaphase or telophase. If these connections become too strong, chromatids might break at or near the points of connection at anaphase. These breaks occurred here at the same point in the sister chromatids, giving rise to fragments of chromosome-like structure [31,39].

It seems reasonable to suggest that Al is either affecting indirectly some metabolic process associated with cell division, or that it has its effect during DNA replication in interphase [32]. Al has been found to inhibit cell division and to be associated with DNA in several plants [44,45]. Matsumoto indicated that one toxic function of Al in rapidly growing pea roots was the binding of Al to DNA regions unmasked with chromosomal proteins in nuclei causing the condensation and stabilization of chromatin structure and thereby reducing the template activity. Thus, cell division at root tips is inhibited by Al toxicity [46]. Recent studies have demonstrated that Al toxicity is associated with mitochondrial dysfunction and the production of reactive oxygen species (ROS) in plant cells [47,48]. The attack of ROS to purine-, pyriminide-bases, and deoxyribose in DNA can cause DNA single and double strand breaks, which may increase the probability of chromosomal aberrations formation.

MTs, one key components of the eukaryotic cytoskeleton, play a central role in diverse activities such as cell division, cell expansion, cell differentiation and intracellular organization and motility [49,50]. Cytoskeletal network of plant cell is formed by the integrated arrays of MTs, actin filaments, intermediate filaments, microtubule- and actin-related proteins and others [51,52]. Al, in micromolar concentrations, delays microtubule (MT) re-organization and MT-mediated chromosome movement during mitosis in the root tip cells of *Triticum turgidum* [53,54]. Frantzios et al. reported that Al affected the mechanisms controlling the organization of the microtubule cytoskeleton as well as tubule polymerization, which delayed microtubule disassembly during mitosis, resulting in the disorder of chromosome movements carried out by the mitotic spindle [53]. The findings of this study showed that the Al ions directly or indirectly affected the dynamic condition of MTs and disturbed the organization and function of the mitotic apparatus, resulting in disordering or inhibition of chromosome movement. Al induced the formation of abnormal microtubule (MT) arrays, consisting of discontinuous wavy MTs or short MT fragments at the cell periphery (Figure 4). With increased Al concentration and prolonged duration of treatment, MT organization and function of the mitotic spindle and phragmoplast were severely disturbed (Figure 5). This study revealed that the MT cytoskeleton was a target site of Al toxicity in mitotic root-tip cells of *P. massoniana*. The structural changes have been attributed to depolymerization of the cortical MTs or to extensive cell-specific depolymerization or to reorganization and stabilization of MTs and actin filaments [55].

In this investigation, the contents of Mg decreased in roots with increasing Al. Al transport through the Mg channel is also likely because Mg transport is strongly inhibited by Al due to the similar diameter of Al and Mg [56]. Al can bind to nucleoside triphosphates approximately 10^7^ times better than Mg, and the rate of hydrolysis for Al-ATP or Al-GTP complexes is considerably lower than that for the physiological Mg complex (10^5^ times slower), supporting the hypothesis that toxicity is a result of Al ions displacement of Mg ions from nucleoside di- or triphosphate complexes [57]. As the replacement of Mg ions by Al ions during tubulin polymerization *in vivo*, MTs with atypical properties may be formed [58].

### Toxic effects of Al on nucleoli

The silver staining technique has been widely applied in cytological studies aimed at understanding the nucleolar cycle and nucleolar organization in both animals and plants. NORs are defined as nucleolar components containing a set of argyrophilic proteins, which are selectively stained by silver methods [59,60]. Changes of argyrophilic proteins in nucleoli can be showed specifically using silver staining method. Evidence from the present investigation revealed that the nucleolar material in some root tip cells did not completely disappear during metaphase under Al stress (Figure 6), which was similar to the phenomenon observed by Qin et al. [61]. Generally, persistent nucleoli do not occur during normal mitosis. Vostrikova and Butorina [62] indicated an increased number of persistent nucleoli cells increased nucleolar activity. Sheldon et al. [63] studied a series of embryonal carcinoma lines and found them to exhibit nucleolar persistence during mitotic metaphase and anaphase, supposing that rRNA synthesis continues in persistent nucleoli, which means increased biosynthetic activity and more protein production. It was reported that the birch seedling in response to anthropogenic stress (heavy metals, smoke, dust et al.) revealed persistent nucleolus in some cells during mitotic metaphase [62]. It was demonstrated that Cd and Cr (VI) could induce persistent nucleoli in root tips of *Vicia faba* [64] and *Allium cepa* [37]. Thus, it may be hypothesized that the phenomenon persistent nucleolus is an adaptive response to stress induced by Al.

The Al-induced phenomena observed in this work were that some tiny particles containing argyrophilic proteins were scattered in the nucleus of root tip cells and leached out from the nucleus to the cytoplasm (Figure 7). These effects of Al on nucleoli are more or less the same as those reported by Fiskesjö [65,66], Liu and Jiang [67], Zhang et al. [64] and Qin et al. [61]. However, they did not indicate what kinds of proteins involved in those reports. The recent studies reported that the argyrophilic proteins were B23 and C23 in *Allium cepa* [68] and in *Hordeum vulgare* (unpublished) by indirect immunofluorescence and western blotting. Evidence in this investigation indicated that Al could induce the three proteins extruded from nucleoli to cytoplasm (Figures 8, 9 and 10), which confirmed the findings reported by Qin et al. [68]. The nucleolus is the most prominent substructure in the cell nucleus, which is a very dynamic structure and form around the rDNA loci [69]. It plays important roles in the regulation of many fundamental cellular processes, including cell cycle regulation, apoptosis, telomerase production, RNA processing, monitoring and response to cellular stress [70,71]. B23 and C23 belong to the two major Ag-NOR proteins [72,73]. However, fibrillarin is distinguished from them by its lack of affinity for silver staining [74]. It has a molecular mass of 41 kDa and is present both in animal and plant cells and is required for multiple events leading to rRNA maturation and ribosome subunit assembly [75]. So we suggest that Al may also have toxic effects on other kinds of nucleolar proteins besides argyrophilic and acidic nucleolar proteins. More studies, however, are required in this direction.

Western blot analysis of the expression of the three proteins showed that Al could induce significant bulking of expression (Figure 11). The changes of localization and expression of the three proteins can be used as valuable and early markers in cellular changes induced by Al for the evaluation of metal contamination. Nevertheless, more studies including the interaction of nucleolus proteins and the molecular mechanism of Al stress will be required to be clarified.

## Conclusion

Based on the information provided in this article, it is concluded that (1) The accumulation of Al ions primarily was in roots of *P. massoniana*, and small amounts of Al were transported to aboveground. (2) Al could disturb cell division inducing chromosome aberration. (3) In the presence of Al, the abnormal MT arrays were formed, and MT organization and function of the mitotic spindle and the phragmoplast were severely disturbed. (4) Persistent nucleoli existed during metaphase. (5) Al could affect nucleoli, inducing nucleolar particles containing argyrophilic proteins leached out from the nucleus to the cytoplasm. (6) Evidence confirmed that these proteins were B23, C23 and fibrillarin. (7) Al could induce over-expression of the three major nucleolar proteins*.*

## Methods

### Al determination

#### Culture condition and Al treatment

Healthy and equal-sized seeds were chosen from *P. massoniana*. The seeds were soaked in tap water for 48 h. Then, they were transferred to a tray lined with wet gauze and germinated in light incubator at 26°C. The seedlings with about 4.5 cm were grown in containers with 2 L Hoagland’s nutrient solution (pH 4.0) adding different concentrations of Al solutions (10^−5^, 10^−4^, 10^−3^ and 10^−2^ M) for 40 d respectively in a greenhouse where relative humidity (60%) and supplementary lighting (14 h photoperiod) were controlled. The Al was provided as aluminum chloride (AlCl_3_). The Al solutions were prepared in deionized water, and were added to the modified half Hoagland’s nutrient solution [61] containing 5 mM Ca (NO_3_)_2_, 5 mM KNO_3_, 1 mM KH_2_PO_4_, 1 mM MgSO_4_, 50 μM H_3_BO_3_, 10 μM FeEDTA, 4.5 μM MnCl_2_, 3.8 μM ZnSO_4_, 0.3 μM CuSO_4_, and 0.1 μM (NH_4_)_6_Mo_7_O_24_ (pH = 4.0). The half Hoagland’s nutrient solution without Al was used for control plants. The solutions were aerated by pumps, which connected the containers with pump lines. The nutrient solutions were changed regularly every 10 days. The seedlings from each treatment were harvested after 40 d of incubation for Al determination.

#### Sampling procedure and Al determination

Ten seedlings from each treatment and control were harvested based on uniformity of size and colour (removing the greatest and the smallest seedlings and then selected randomly) at the end of each time interval (10 d). The seedlings were removed from solution and washed thoroughly with running tap water for 30 min and then with deionized water to remove traces of nutrients and Al ions from root surfaces. Seedlings were divided into roots, leaves and stems. The samples were dried to a constant weight (for 3 d at 45°C, for 1 d at 80°C, and for 12 h at 105°C). All dried plant samples were prepared using a wet-digestion method [62]. The contents of Al, Mg, Mn and Fe were determined with inductively coupled plasma atomic emission spectrometry (ICP-AES) (Leeman Labs Inc., New Hampshire, USA) after dry-ashing [63].

### Cytological study

#### Culture condition and Al treatment

Healthy and equal-sized seeds were chosen from *P. massoniana*. The seeds were soaked in tap water for 48 h before starting the experiment. Then they were transferred to plastic containers, which in the bottom has wet gauze to germinate at 26°C for several days. Then the roots reaching about 0.6 cm length were treated in Petri dishes with different concentrations of Al solutions (10^−5^ to 10^−2^ M) for 24, 48 and 72 h. The test liquids were changed regularly every 24 h. Control roots were maintained in distilled water. The Al was provided as aluminum chloride (AlCl_3_). The length of roots were observed, measured and recorded at the end of each time interval (324 h).

#### Staining method

Ten root tips from control and seedlings of *P. massoniana* treated with Al were cut and fixed in 3 parts 95% ethanol: 2 parts acetic acid for 2 h and hydrolyzed in 5 parts 1 M hydrochloric acid: 3 parts 95% ethanol: 2 parts 99.8% acetic acid for 10 min at 60°C. For the observation of changes in cell division, ten root tips were squashed in Carbol Fuchsin solution [64]. For the observation of changes in nucleolus, ten root tips were cut and squashed in 45% acetic acid, dried, and after 2 days stained with silver nitrate [53].

#### Indirect immunofluorescent microscopy

Meristematic zones of root tips from control and seedlings of *P. massoniana* treated with Al were cut and fixed with 4% (w/v) paraformaldehyde in phosphate-buffered saline (PBS, pH 7.0) for 1.5 h in darkness at room temperature and then they were washed with the same buffer. Meristematic cells were digested with a mixture of 2.5% cellulose and 2.5% pectolase at 37°C and then washed in PBS for three times. They were squashed on slides and extracted in freshly prepared 1% (v/v) Triton X-100 in PBS when slides dried. After three washings in PBS, the cells were subsequently incubated with mouse primary antibodies respectively against B23, fibrillarin, C23 or tubulin for 1 h at 37°C or at 4°C overnight in a moist, sealed chamber. After washing (3 × 10 min) in PBS, the cells were incubated with secondary antibodies for detection of the primary antibodies for 45 min in darkness at 37°C. After repeated washing in PBS, nuclei were stained with 4′, 6-diamidino-2-phenylindole (DAPI, Sigma) at a final concentration of 1ug per 1 ml for 15 min at room temperature. After washing (3 × 10 min) in PBS, the cells were mounted in antifade mounting medium. The slides were stored at 4°C in the dark until observed. The immunofluorescent specimens were examined with a Nikon ECLIPSE 90i confocal laser scanning microscope. An exciter at 488 nm and a barrier at 590/50 nm, an exciter at 543 nm and a barrier at 650 nm, and an exciter at 408 nm and a barrier at 515/30 nm were used for FITC, TRITC and DAPI staining, respectively. Image recording was done with proper software (EZ-C1 3.80) according to the manufacturer’s instructions. Images were processed with Image-Pro Plus 6.0 and Photoshop CS3.

Antibodies used in this study were as follows:

- B23: primary antibody: a mouse monoclonal antibody to B23 (Sigma, B0556) at dilution 1:100; secondary antibody: FITC-conjugated goat anti-mouse IgGs (Sigma, F9137) at dilution 1:50. FITC was used for the detection of signal.
- Fibrillarin: primary antibody: a mouse monoclonal antibody to fibrillarin (Santa, SC-81273) at dilution 1:100; secondary antibody: FITC-conjugated goat anti-mouse IgGs (Sigma, F9137) at dilution 1:50. FITC was used for the detection of signal.
- C23: primary antibody: a mouse monoclonal antibody to C23 (Santa, SC-8031) at dilution 1:100; secondary antibody: TRITC-conjugated goat anti-mouse IgGs (Sigma, T5393) at dilution 1:50. TRITC was used for the detection of signal.
- Microtubulin: primary antibody: mouse monoclonal anti-a-tubulin antibody (Sigma T-9026) at diluted at 1:100: secondary antibody: FITC-conjugated goat anti-mouse IgGs (Sigma, F9137) at dilution 1:50. FITC was used for the detection of signal.

#### Western blotting

Root tips from control and seedlings treated with 10^−2^ M Al for 72 h were homogenized respectively in a pestle and mortar with liquid nitrogen and then the samples were solubilized with chilled extraction buffer (50 mM Tris–HCl (pH 7.8), 10 mM MgCl_2_, 20 mM β-mercaptoethanol, 1.0 mM EDTA, 8% glycerol) adding protease inhibitor cocktail set VI (Merck, 539133). After vortexing for 1 min at room temperature, the homogenates were kept on ice for 30 min, and then centrifuged at 12,000 rpm at 4°C for 5 min. The mixture of supernatant and 1 × laemmli buffer (62.5 mM Tris–HCl (pH 6.8), 5% β-mercaptoethanol, 2% SDS, 10% glycerol, 0.001% bromophenol blue) was boiled at 100°C for 5 min [65] and then was subjected to 12% SDS-PAGE electrophoresis and the separated proteins were wet blotted onto 0.45 μm PVDF transfer membrane (Millipore, IPVH00010) at 4°C. Blots were blocked for 2 h with 5% (w/v) non-fat milk in TBST buffer at room temperature with shaking. Primary antibody mentioned above against the indicated protein was diluted in TBST buffer (B23, 1:4000; Fibrillarin, 1:4000; C23, 1:3500). Anti-β actin monoclonal antibody (Abmart, P30002) was used for the internal control. The soaked PVDF membrane was subsequently incubated with primary antibody for 2.5 h at room temperature on the rocker platform and then washed with TBST buffer two times for 10 min each and TBS buffer one time for 10 min. The HRP-conjugated secondary antibody (Promega, W4021) diluted 1:7000 in TBST buffer was added for 2 h. After another three washes, the blots were detected using the ECL technique (Millipore, WBKL S0100) and exposed to the X-ray film. The intensities of the bands in the film were measured by software “Quantity One” (Bio-Rad). During the experiment, care was taken to prevent membrane from drying.

#### Statistical analysis

Each treatment was replicated 3 times for statistical validity. SPSS computer software was used for statistical analyses (SPSS Japan Inc., Shibuya, Tokyo, Japan) and SigmaPlot 8.0 software was used for mapping. Any differences between treatments were determined using one-way analysis of variance (ANOVA), and scored as significant if (*P* < 0.05). The means and standard errors of the means mean ± SE are reported. For Western blotting statistical analysis, independent-samples *t*-test was used to determine the significance at *P* < 0.05 using SPSS 15.0 version for Windows software.
